# Application of an Interpretable Machine Learning for Estimating Severity of Graves’ Orbitopathy Based on Initial Finding

**DOI:** 10.3390/jcm12072640

**Published:** 2023-04-01

**Authors:** Seunghyun Lee, Jaeyong Yu, Yuri Kim, Myungjin Kim, Helen Lew

**Affiliations:** 1 Department of Ophthalmology, Konyang University, Kim’s Eye Hospital, Myung-Gok Eye Research Institute, Seoul 07301, Republic of Korea; 2 Department of Biomedical System Informatics, Yonsei University College of Medicine, Seoul 03722, Republic of Korea; 3 Department of Ophthalmology, Bundang CHA Medical Center, CHA University, Seongnam 13496, Republic of Korea

**Keywords:** graves’ orbitopathy, severity, muscle predominant type, risk prediction

## Abstract

(1) Background: We constructed scores for moderate-to-severe and muscle-predominant types of Graves’ orbitopathy (GO) risk prediction based on initial ophthalmic findings. (2) Methods: 400 patients diagnosed with GO and followed up at both endocrinology and ophthalmology clinics with at least 6 months of follow-up. The Score for Moderate-to-Severe type of GO risk Prediction (SMSGOP) and the Score for Muscle-predominant type of GO risk Prediction (SMGOP) were constructed using the machine learning-based automatic clinical score generation algorithm. (3) Results: 55.3% were classified as mild type and 44.8% were classified as moderate-to-severe type. In the moderate-to-severe type group, 32.3% and 12.5% were classified as fat-predominant and muscle-predominant type, respectively. SMSGOP included age, central diplopia, thyroid stimulating immunoglobulin, modified NOSPECS classification, clinical activity score and ratio of the inferior rectus muscle cross-sectional area to total orbit in initial examination. SMGOP included age, central diplopia, amount of eye deviation, serum FT4 level and the interval between diagnosis of GD and GO in initial examination. Scores ≥46 and ≥49 had predictive value, respectively. (4) Conclusions: This is the first study to analyze factors in initial findings that can predict the severity of GO and to construct scores for risk prediction for Korean. We set the predictive scores using initial findings.

## 1. Introduction

The complex pathogenesis arising from the interaction of endogenous factors and environmental triggers, diversity, and ethnic differences in manifestations of Graves’ orbitopathy (GO) has made it difficult to tailor therapies for individual patients [1,2,3,4]. Several factors, including sex, age, and smoking history, are relevant, of which the role of smoking has been clearly elucidated [5,6,7]. The severity of ophthalmopathy has been shown to be positively associated with advanced age and male sex [8,9].

The epidemiological and clinical features for white patients with GO have been well illustrated, but there is a paucity of literature on GO in Asian populations. Li et al. investigated the characteristics of patients with moderate-to-severe GO in China [10]. Asians and Westerners have many differences in orbital parameters, so using the same standard for GO evaluation in one ethnicity will result in incorrect evaluations in others [11,12,13].

In 2018, the European Group on Graves’ Orbitopathy (EUGOGO) identified four independent determinants as predictors of GO development in patients with newly diagnosed Graves’ disease (GD): clinical activity score (CAS), thyrotropin binding inhibiting immunoglobulin (TBII), current smoking, and longer duration of hyperthyroid symptoms [14]. In addition, they constructed a predictive score, Prediction of Graves’ Orbitopathy (PREDIGO), based on these four independent variables. From an ophthalmological viewpoint, clinical aspects and progression of GO range from mild type requiring only conservative treatment to severe type requiring invasive treatment, such as strabismus surgery, surgical orbital decompression, or eyelid surgery. Therefore, based on initial ophthalmic findings, it is very difficult but important to predict to which type the patient will progress. According to Nunery’s classification of extraocular movements, patients with GO having normal ocular motility and predominant lipogenic change were classified as type I, whereas patients with significant restrictive myopathy and diplopia within 20° of the primary position were classified as type II [15]. Type II disease is also called muscle predominant type, restrictive myopathy and infiltrative type of GO. Most GO patients with moderate to severe type have both inflammation of muscles and increased fat, very rarely distinctly. However, we could also see the two types of GO patients who present more prominent proptosis or restrictive strabismus due to EOM involvement in the clinic.

As some symptoms of GO are irreversible and can result in a pronounced loss of quality of life, the condition can be explained to the patient in advance with early commencement of active treatment [16,17]. In many cases, moderate-to-severe type GO has an important effect on quality of life. In particular, diplopia related to enlargement of extraocular muscles is difficult to treat, leading to functional disability [18]. There are many methods to evaluate the activity and severity of GO, such as the CAS and the modified NOSPECS classification, respectively. However, no tools are available to evaluate all findings comprehensively.

Therefore, we classified the disease type according to the initial eye manifestations and the treatment they received at the time of final evaluation retrospectively and constructed a predictive score that can comprehensively predict the moderate-to-severe type of GO for Korean subjects. In addition, we attempted to identify factors at initial examination that were significantly different between patients requiring active treatment for diplopia related to the enlargement of extraocular muscles and those who did not, in the moderate-to-severe type group, and to construct a predictive score.

## 2. Materials and Methods

### 2.1. Study Design and Setting

We performed a retrospective cohort study of patients treated at the Department of Ophthalmology of Bundang CHA Medical Center, Republic of Korea. This study was approved by Bundang CHA Medical Center Institutional Review Board (IRB Number: 2021-07-054). All data were de-identified and the requirement for informed consent was waived due to the retrospective nature of the study. The study was performed in accordance with the tenets of the Declaration of Helsinki.

### 2.2. Study Population

Patients referred to the Department of Ophthalmology of Bundang CHA Medical Center for evaluation of GO between October 2002 and February 2021 were screened for inclusion in the study. The inclusion criteria were diagnosis of GO and follow-up at both endocrinology and ophthalmology clinics of our hospital for at least 6 months. Patients with an incomplete set of laboratory measurements, CT images, or other parameters for assessment of GO were excluded.

### 2.3. Outcome

The primary outcomes were determination of which initial ophthalmic findings were significantly different between types of GO. The disease type was classified at the time of final evaluation. Mild type was defined as the modified NOSPECS scores ≤ 5 and clinical symptoms necessitating only conservative treatment. If the mild type of clinical manifestation did not progress for at least 6 months, the severity of the disease was still considered as mild type. Moderate-to-severe type was defined as clinical symptoms requiring treatment other than conservative treatment at least once. These patients were divided into fat predominant and muscle predominant types. Fat predominant type was defined as presenting with clinical symptoms such as proptosis and lid retraction requiring active treatment, but no definite external ocular muscle (EOM) involvement. The most important factor is that these symptoms must be correlated with a definite increase in fat volume on CT. Muscle predominant type was defined as EOM involvement leading to diplopia within 30° of field of view on binocular single vision test (BSV test) and requiring treatment.

The treatments for moderate-to-severe type include periorbital injection using triamcinolone acetonide, Botulinum neurotoxin type A (BoNT-A) and fillers, lid surgery, systemic corticosteroids, radiotherapy of the orbits, surgical orbital decompression, and strabismus surgery. For fat predominant type, periorbital injection (triamcinolone acetonide, BoNT-A, fillers), lid surgery, systemic corticosteroids and surgical orbital decompression were considered. In the acute phase, periorbital injection (triamcinolone acetonide, BoNT-A, fillers) was considered as an option for patients with severe eyelid abnormalities such as lid retraction and entropion that interfere with daily life. In the chronic phase, lid surgery was performed according to the type of symptoms. Systemic corticosteroids were primarily considered for patients with CAS 4 or higher and surgical orbital decompression was considered for patients with exophthalmos of 3 mm or more in the chronic phase. For muscle predominant type, systemic corticosteroids, radiotherapy of the orbits, BoNT-A injection to EOM and strabismus surgery were taken. First, radiotherapy was primarily considered for patients with diplopia and strabismus corresponding to muscle enlargement in the acute phase, but systemic corticosteroids were also selected according to the patient’s preference or general conditions. For chronic patients, BoNT-A injection to EOM or strabismus surgery was considered, depending on the degree of strabismus or diplopia.

We developed scores, i.e., Score for Moderate-to-Severe type of Graves’ Orbitopathy risk Prediction (SMSGOP) and Score for Muscle predominant type of Graves’ Orbitopathy risk Prediction (SMGOP), to comprehensively predict the severity and type of GO, based on the secondary outcomes.

### 2.4. Putative Determinants

Putative determinants for severity of GO were assessed at initial eye examination. They included age, sex, follow-up duration of GD and GO, interval between diagnosis of GD and GO, medical history (hypertension, diabetes mellitus, cerebrovascular disease, autoimmune diseases, and statin use), smoking behavior (never smoker, ex-smoker, current smoker), history of GD treatment (radioactive iodine therapy (RAI) or thyroidectomy), CAS, the modified NOSPECS classification, proptosis by Hertel exophthalmometry (Oculus; Oculus Optik Geraete, Wetzlar, Germany), type and amount of eye deviation by HESS test, presence of central diplopia by BSV test, orbital computed tomography (CT) to measure cross-sectional area of each external ocular muscle except the inferior oblique muscle, orbital fat, and total orbit at 4 mm behind the eyeball. Each of external ocular muscle and orbital fat were calculated as a ratio to the total orbit. Biochemical severity of GD, i.e., thyroid stimulating hormone (TSH), free triiodothyronine (FT3), and free thyroxine (FT4) levels, and immunological severity, i.e., thyroid stimulating hormone receptor antibodies (TSH-R Ab), thyroglobulin antibody (TG Ab), thyroid peroxidase antibody (TPO Ab), and thyroid stimulating immunoglobulin (TSI), were also evaluated.

We used variable immunoassay kits: Atellica IM Free Thyroxine (Siemens Healthcare Diagnostics Inc., Malvern, PA, USA), (RRID:AB_2895179) for free Thyroxine (FT4), Atellica IM Thyroid Stimulating Hormone 3-Ultra (Siemens Healthcare Diagnostics Inc., Malvern, PA, USA), (RRID:AB_2895183) for Thyroid Stimulating Hormone 3-Ultra(TSH3-UL), ARCHITECT Free T3 Reagent Kit (ABBOTT, Wiesbaden, Germany), (RRID:AB_2885163) for Free T3, Elecsys Anti-TSHR_P (Roche Diagnostics GmbH, Vienna, Austria), (RRID:AB_2801453) for TSH receptor Ab, Elecsys Anti-Tg_P (Roche Diagnostics GmbH, Vienna, Austria), (RRID:AB_2894922) for Thyroglobulin Ab, Elecsys Anti-TPO (Roche Diagnostics GmbH, Vienna, Austria), (RRID:AB_2631044) for Thyroid peroxidase Ab, Thyretain^TM^ (Diagnostic Hybrids, Inc., Athens, OH, USA) for TSI.

### 2.5. Statistical Analysis

Some continuous variables were transformed into categorical variables for ease of interpretation and to deal with nonlinearity. Serum TSH, FT4, and FT3 levels were categorized into three groups: below the normal range, normal range, and above the normal range. TSH-R Ab, TG Ab, TPO, and TSI were categorized into two groups: negative and positive.

In the descriptive summaries for the demographic table, categorical variables are shown as the frequency (percentage) and continuous variables are reported as the mean (standard deviation). Comparisons were performed with the *t* test and chi-square tests at the 5% significance level. The data were analyzed using R software, version 3.5.3 (R Foundation for Statistical Computing, Vienna, Austria).

### 2.6. AutoScore

We implemented the AutoScore framework (version 1.0.0), a machine learning-based automatic clinical score generation algorithm developed by Nan and Feng [19,20]. AutoScore combines machine learning and logistic regression for variable ranking and coefficient estimation, integrates data manipulation, such as categorization, and automates the development of parsimonious sparse-score risk models for the outcomes.

AutoScore was used to select the most discriminative variables from all candidate variables. Parsimony plots (i.e., model performance vs. complexity) based on the validation set were used to determine the choice of variables. We conducted bootstrap variable selection to solve the problems of initialization and small data. We chose several variables that showed more than a certain level of improvement in performance. For defining “certain level of improvement”, we made 50 boot samplings. For each 50 Random Forest feature importance, we measured the improvement of AUROC as we added the variables one by one from most important variables list. If more than half was obtained (25 here) of improvement in AUROC after adding the variable, we selected those variables as final features. These selected variables highlighted the severity of GO on initial examination. The performance did not improve markedly when more variables were added to the scoring model. Predictive performances of the SMSGOP and SMGOP scores are reported based on the testing cohort with 95% confidence interval (CI), and validation of the score was visualized with binned scatter-plot and non-linear regression. The data were randomly split into two cohorts: a training cohort (80%) for development and a validation cohort (20%) for evaluation. Supplementary Table S1 shows that there was no statistically significant difference between training and validation cohort under 5% of significance level. We used the autoscore term “risk” meaning, or equivalent to, “possibility”, and the “risk” was quantified as a score.

## 3. Results

### 3.1. Subtype

**Figure 1:**
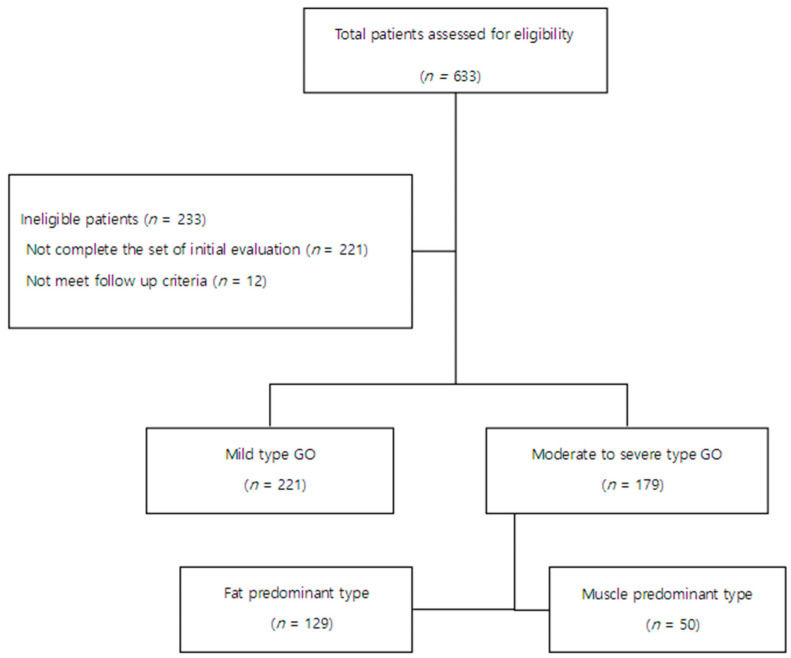
Flowchart showing trial profile. GD; Graves’ disease, GO; Graves’ Orbitopathy.

A total of 633 patients referred to the Department of Ophthalmology of Bundang CHA Medical Center for evaluation of GO between October 2002 and February 2021 were assessed for eligibility. Among them, 221 did not complete the initial evaluation for GO, and 12 did not meet the follow-up criteria (Figure 1). Finally, 400 patients diagnosed with GO and followed up at both endocrinology and ophthalmology clinics of our hospital for at least 6 months were included in the study.

Of the 400 patients included in the study, 221 (55.3%) were classified as having mild type disease and 179 (44.8%) as moderate-to-severe type disease requiring active treatment. Among the moderate-to-severe type subgroup, 129 (32.3%) were classified as fat predominant type and 50 (12.5%) as muscle predominant type. Their characteristics at initial examination are listed in Table 1. Patients who developed moderate-to-severe type GO tended to be older, had hypertension, smoking history (ex-smoker or current smoker), had normal or low serum FT4 level, and were positive for TSI. They had a higher CAS and the modified NOSPECS classification, showed higher rate of vertical and mixed eye deviation, a large amount of eye deviation, central diplopia, and had a higher ratio of cross-sectional area of inferior rectus muscle to total orbit. Patients who developed muscle predominant type GO tended to be older, male, had hypertension and autoimmune diseases, history of statin use, normal serum FT4 level, higher CAS and the modified NOSPECS classification, presented higher rate of eye deviation, a large amount of eye deviation, central diplopia, and a higher ratio of cross-sectional area of inferior rectus muscle and lower ratio of cross-sectional area of lateral rectus muscle to total orbit than patients with fat predominant type.

### 3.2. Moderate-to-Severe Type GO and SMSGOP Score

**Figure 2:**
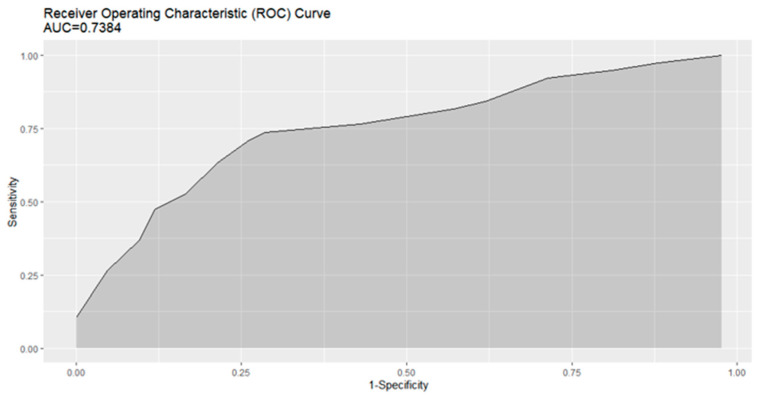
Receiver-operator curve with confidence intervals of the SMSGOP for predicting moderate to severe type of Graves’ orbitopathy in patients with Graves’ hyperthyroidism. SMSGOP; The Score for Moderate to Severe type of Graves’ Orbitopathy Risk Prediction.

We chose six variables as the parsimonious choice for severity of GO and they achieved a good balance in the parsimony plot, i.e., age, central diplopia, TSI, the modified NOSPECS classification, CAS, and ratio of cross-sectional area of inferior rectus muscle to total orbit. These variables highlighted the severity of GO on initial examination. Addition of more variables to the scoring model did not markedly improve the performance. Table 2 shows the SMSGOP based on the six variables. Points were assigned to the presence or absence of each variable, and the sum of the points provided a numerical predictive score ranging from 0 to 101. The performance of the predictive score was evaluated in the testing cohort. Supplementary Figure S1A shows the distribution of episodes at different score intervals, which had a near-normal distribution. As shown in Supplementary Figure S1B, the rate of moderate-to-severe type GO increased along with increasing score in the testing cohort. For predictive score, most patients had a risk score between 30 and 70, and few had scores <20. Scores ≥46 had some predictive value for moderate-to-severe type GO. The predictive score had sensitivity of 0.737 (95% CI: 0.605–0.868), specificity of 0.714 (0.571–0.833), positive predictive value of 0.7 (0.595–0.813), and negative predictive value of 0.75 (0.651–0.857). The area under the receiver operating characteristic (ROC) curve (AUC) of the predictive score was 0.738 (0.627–0.850) (Figure 2).

### 3.3. Muscle Predominant Type GO and SMGOP Score

**Figure 3:**
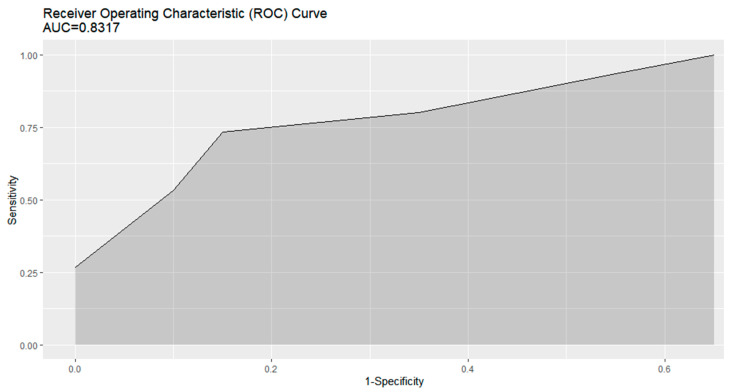
Receiver-operator curve with confidence intervals of the SMGOP for predicting muscle predominant type of Graves’ orbitopathy in patients with moderate to severe type of Graves’ orbitopathy. SMGOP; The Score for Muscle predominant type of Graves’ orbitopathy Risk Prediction.

We chose five variables as the parsimonious choice for muscle predominant type GO: age, central diplopia, amount of eye deviation, serum FT4 level, and interval between diagnosis of GD and GO. Table 3 shows the SMGOP. The sum of the points provided a numerical predictive score ranging from 0 to 101. We also evaluated the performance of the predictive scores in the testing cohort. Supplementary Figure S2A shows the distribution of episodes at different score intervals, which showed a near-normal distribution. As shown in Supplementary Figure S2B, the rate of muscle predominant type GO increased along with increasing score in the testing cohort. Scores ≥49 had some predictive value for muscle predominant type GO. The predictive score showed sensitivity of 0.733 (95% CI: 0.467–0.933), specificity of 0.85 (0.7–1.0), positive predictive value of 0.8 (0.611–1.0), and negative predictive value of 0.818 (0.682–0.947). The AUC of the predictive score was 0.832 (0.696–0.967) (Figure 3).

## 4. Discussion

GO has unique characteristics in different ethnic groups, and its clinical presentations vary according to age, sex, smoking status, and other external factors [4]. In this study, 55.3% of patients with GO had mild type and 44.8% had moderate-to-severe type. The GO type was determined by reviewing the previous patient records when GD was stable for more than 6 months. In the moderate-to-severe type subgroup, 129 patients (32.3%) had fat predominant type and 50 (12.5%) had muscle predominant type. There has been controversy in the reported prevalence and characteristics of GO in Asian patients [21]. Not only the severity but also the prevalence of GO in GD patients were previously reported and discussed in the nation-wide multicenter study in Korea. This showed that GO was present in about 17% of GD patients, which was quite low compared with the British studies, in which the prevalence of GO (NOSPECS score ≥ 2) was reported to be 52% in a GD cohort of 2405 patients [1,22]. Some studies have suggested that ethnic differences in prevalence are related to different smoking rates [21]. In another comparative study on the prevalence of GO comparing European and Asian populations, the prevalence was 42% in Europeans compared to 7.7% in Asians, and the overall risk for Europeans for developing GO was 6.4 times higher than for Asians. In this group, the smoking rate was 61.2% in Europeans and 23% in Asians. The smoking factor is known to be a risk factor in Europeans; however, the role of smoking in the Asian population is complex and warrants further studies. Multifactorial etiologies could affect the difference in GO between diverse ethnic groups.

Li Q et al. analyzed the clinical features of patients with moderate-to-severe GO in China and reported that the severity of GO was significantly associated with male sex, older age, smoking, family history of thyroid disease, and degree of proptosis [10]. In addition, they reported that the female-to-male ratio and mean value of exophthalmos were significantly lower in Chinese patients compared with white patients. In addition, the inferior and superior rectus muscles were the most common extraocular muscles involved in Chinese patients and, therefore, lower eyelid retraction should be included in the diagnostic criteria in Asian patients. We not only analyzed the clinical features of patients with moderate-to-severe GO but also performed a comparison with mild type at initial examination and proposed predictive scores for moderate-to-severe type GO. Associations between the factors included in the scores and GO were demonstrated in previous studies [8,9,23,24,25]. However, this is the first study to present a predictive score with these factors for use at initial examination in Korea. Choi JH et al. reported that a high titer of TSAb may be predictive of a poor prognosis for muscle predominant type [26]. They divided the patients with muscle predominant type of GO into the improved or not-improved groups, and they showed that patients with muscle predominant type who had higher pre-treatment TSAb titers showed poorer responses to treatment.

The classification of GO as mild or moderate has been carried out in most of the literature based on EUGOGO criteria. The classification based on EUGOGO criteria was for establishing treatment guidelines, and in our study, the disease type was decided by the severity, based on the modified NOSPECS score system at the time of final evaluation. In other words, patients were retrospectively classified according to the eye manifestations and the type of treatment they received. This classification helps predict the disease progression, related to severity and need of medical intervention based on the initial eye manifestations.

We selected variables as predictors of moderate-to-severe type GO to construct a predictive score, i.e., age, central diplopia, TSI, the modified NOSPECS classification, CAS, and ratio of the cross-sectional area of inferior rectus muscle to the orbit. We used the autoscore term “risk” meaning, or equivalent to, “possibility” and the “risk” was quantified as a score. This predictive score (SMSGOP) has higher values than PREDIGO except for specificity (0.714 vs. 0.75, respectively) [14], and could be a good predictive tool for moderate-to-severe type GO.

At initial presentation, 385 patients showed the mild type and 15 patients showed the moderate to severe type of GO. Among initial ‘mild type’, 219 patients (56.9%) stayed as mild type, but 166 patients (43.1%) progressed to the moderate to severe type. Among initial ‘moderate to severe’, 13 patients (86.7%) stayed as moderate to severe type, but 2 patients (13.3%) turned to the mild type. So, 58% of patients would stay at the initial type of GO, but 42% of the patients progress to another type of GO, based on this SMSGOP model during the follow up period at least 6 months.

In terms of each component of the predictive scores, the modified NOSPECS classification of 4 was associated with the highest corresponding risk (quantified as score). Higher CAS and modified NOSPECS classification tended to correspond to a higher predictive score. CAS <3 corresponded to a score of 0, while CAS > 3, indicating the active phase, was associated with the highest score. It should be noted that, the more severe and active GO was from the time of first evaluation, the more likely it was to develop into moderate-to-severe type GO. The score was the highest for age 51–64 years and second highest for age 28–51 years. The incidence of GD is known to show two age peaks in the fifth and seventh decades of life, with a mean age of about 43 years [27]. This is consistent with our findings. Therefore, we confirmed that more severe GO occurs at the time when the incidence of GD is highest. The risk is very low in children and adolescents, and zero in people in their 20s. The presence of central diplopia at initial examination had the highest score among all variables. Therefore, it is a key factor predicting moderate-to-severe type GO, and patients with central diplopia on initial examination should be treated more actively and with greater care. In the EUGOGO classification, there is also an ‘inconstant or constant diplopia’ item in the criteria for Moderate-to-severe GO. This finding is in accordance with the EUGOGO classification. Positivity for TSI was also a meaningful factor for prediction of severity of GO. Ponto KA et al. reported that TSI showed significant associations with activity and severity of GO [28]. Therefore, the increase in risk when TSI is positive can be seen in the same context as the increase in risk with increases in CAS or modified NOSPECS classification. Thus, testing for TSI would be useful in predicting the severity of GO. A high ratio of cross-sectional area of inferior rectus muscle to total orbit at initial evaluation tended to be associated with high scores. Enlargement of the inferior rectus muscle, therefore, appears to occur from the beginning of GO.

Regensburg NI et al. divided GO patients into four groups—with no increases in orbital fat volume (FV) or extraocular muscle volume (MV) (25.3%); with only FV increase (5.3%); with only MV increase (61.1%); and with both FV and MV increases (8.4%)—and reported that increases in MV were present in the largest proportion [18]. Comparison of the groups with and without MV increase indicated that patients with increased MV were older, had more proptosis, more impaired ductions (abduction, adduction, and elevation), more diplopia, and higher TBII titer. Relative to patients without increased FV, patients with increased FV had more proptosis and less diplopia. Du B et al. also reported increases in MV (70%) in the largest proportion of their patients, and MV increase was found to be related to older age, higher TBII titer, more proptosis and, as expected, reduced duction values [29]. These findings differed in many respects from the results of the present study, in which muscle predominant type accounted for only 12.5% of the total patients with GO. These differences may have been because previous studies evaluated MV enlargement based only on imaging examinations, while we defined muscle predominant type as diplopia corresponding to MV enlargement on CT data, which could be an even more functional classification. Similarly, the rates of patients with central diplopia in these groups were not low as 2.3%, 14.7%, and 70%, in the mild type, fat predominant type, and muscle predominant type, respectively. Diplopia was not defined by subjective questionnaire but based on the Goldman binocular single vision test, thus representing true diplopia related to EOM enlargement.

Amount of eye deviation, age, central diplopia, serum FT4 level, and the interval between diagnosis of GD and GO are predictors of muscle predominant type of GO. SMGOP had higher predictive value than SMSGOP, except for lower sensitivity (0.733 vs. 0.737, respectively), and could be a very effective tool for evaluation generally.

In terms of different components of predictive scores, amount of eye deviation <15° had a corresponding risk of 0, while >45° had the highest corresponding score of 43. If the EOM is severely affected leading to a large amount of eye deviation from the beginning of GO, this is a powerful indicator suggesting that the disease will progress to the muscle predominant type. Age ≥ 64 years was associated with the highest risk, with a score of 45. In older patients who have passed the acute phase, fibrotic changes can be assumed to occur in the extraocular muscles, causing irreversible changes and diplopia. The risk increased significantly from 0 to 38, with age > 32 years, so patients in this age group should be monitored carefully. The presence of central diplopia at initial examination was also associated with risk of muscle predominant type disease. When the interval between diagnosis of GD and GO was >10 years, the corresponding risk was 0. GO usually occurs within 2~3 years after GD diagnosis in most patients. However, it could also be interpreted that GO does not occur well in patients with GD prevalence period of more than 10 years, but even if it occurs, the probability of turning into the muscle predominant type is small. Some GD patients have been under anti-thyroid medication for more than 10 years, and they could happen to present GO manifestations at that time or later. Even though 10 years is not the usual time point for GD follow up, it was the meaningful cut-off time point in this study.

In our study, we could obtain CT data for the GD patients with suspected thyroid ophthalmopathy, even with mild symptoms to evaluate the orbit. We presented six variables for moderate to severe type, so even if there is no CT data the other variables could help in predicting it. Five variables were applied for muscle predominant type, but they did not include CT data.

This study may be flawed, because the inclusion criteria and study settings are not equivalent to other studies, since most patients for our study were referred by endocrinologists and general ophthalmologists, and their acute phase could have passed when they finally visited our clinic. This can be inferred by the CAS scores of the patients initially visited. The average CAS scores were 1.2, 2.4, and 3.0 in the mild type, fat predominant type, and muscle predominant type, respectively. It also had some limitations related to its retrospective nature and sample size. Machine learning classification has been proposed in GO previously [30]. In that study, only images for patients with GO were used. Therefore, it was easy to obtain data, and as many as 21,840 images from 1560 patients were used for the study. However, in our study, many data were necessary and those who did not have these records were excluded. Therefore, the number of patients was significantly insufficient and the bootstrap variable selection was performed to solve the problems of initialization and small data. Further prospective studies conducted in multiple centers would yield more reliable results. It is also necessary to find out how each variable acts as a risk factor for severity and type of GO over time, i.e., time-series analysis. This will be analyzed through further study. We will be prepared soon to study the prediction of the activity course of GO patients based on the initial eye examination.

## 5. Conclusions

In conclusion, both SMSGOP and SMGOP are two models of artificial intelligence system for prediction of GO progression when doctors see Graves’ patients, based on the initial eye manifestations. These are very useful for doctors to explain the potential severity to patients and decide follow up and management plans on a regular basis. Therefore, we definitely recommend that doctors, not only ophthalmologists but also endocrinologists, apply the SMGOP model to pay attention to potential severity in GO patients and to treat these patients at the right time in the proper manner, which could avoid functional complications and disfigurement.

## Figures and Tables

**Table 1:** Baseline characteristics at initial examination of 400 patients with Graves’ disease and Graves’ orbitopathy.

	Mild Type GO (n = 221, 55.3%)	Severe Type GO (n = 179, 44.8%)				*p*
		Fat Predominant Type (n = 129, 32.3%)	Muscle Predominant Type (n = 50, 12.5%)	Total	*p*	
Age (years)	37.0 ± 13.3	39.2 ± 12.9	52.6 ± 11.5	42.9 ± 13.9	<0.001	<0.001
Gender					0.008	0.456
Female	170 (76.9%)	102 (57.0%)	29 (16.2%)	131 (73.2%)		
Male	51 (23.1%)	27 (15.1%)	21 (11.7%)	48 (26.8%)		
Duration of follow up for GD (months)	58.4 ± 48.2	62.4 ± 55.7	57.5 ± 55.6	61.0 ± 55.6	0.598	0.62
Duration of follow up for GO (months)	44.7 ± 38.2	49.7 ± 45.1	45.7 ± 43.0	48.6 ± 44.4	0.59	0.354
Interval between diagnosis of GD and GO (months)	16.6 ± 37.2	22.4 ± 46.0	23.3 ± 47.0	22.7 ± 46.2	0.906	0.158
Hypertension	16 (7.2%)	12 (6.7%)	13 (7.3%)	25 (14.0%)	0.008	0.041
Diabetes mellitus	10 (4.5%)	1 (0.6%)	3 (1.7%)	4 (2.2%)	0.119	0.334
Cerebrovascular disease	4 (1.8%)	1 (0.6%)	2 (1.1%)	3 (1.7%)	0.39	1
Autoimmune diseases	5 (2.3%)	0 (0.0%)	3 (1.7%)	3 (1.7%)	0.031	0.954
History of taking statin	21 (9.5%)	14 (7.8%)	13 (7.3%)	27 (15.1%)	0.021	0.12
Smoking behavior					0.084	0.007
Never smoker	204 (92.3%)	111 (62.0%)	40 (22.3%)	151 (84.4%)		
Ex-smoker	3 (1.4%)	6 (3.4%)	7 (3.9%)	13 (7.3%)		
Current smoker	14 (6.3%)	12 (6.7%)	3 (1.7%)	15 (8.4%)		
History of RAI *	4 (1.8%)	3 (1.7%)	2 (1.1%)	5 (2.8%)	0.917	0.749
History of thyroidectomy	4 (1.8%)	6 (3.4%)	3 (1.7%)	9 (5.0%)	1	0.128
fT3					0.121	0.245
Lower	35 (15.8%)	24 (13.4%)	12 (6.7%)	36 (20.1%)		
Normal	94 (42.5%)	56 (31.3%)	24 (13.4%)	80 (44.7%)		
Higher	92 (41.6%)	49 (27.4%)	14 (7.8%)	63 (35.2%)		
fT4					0.03	0.01
Lower	27 (12.2%)	20 (11.2%)	6 (3.4%)	26 (14.5%)		
Normal	88 (39.8%)	60 (33.5%)	34 (19.0%)	94 (52.5%)		
Higher	106 (48.0%)	49 (27.4%)	10 (5.6%)	59 (33.0%)		
Thyroid Stimulating Hormone (TSH)					0.169	0.123
Lower	164 (74.2%)	89 (49.7%)	27 (15.1%)	116 (64.8%)		
Normal	39 (17.6%)	28 (15.6%)	16 (8.9%)	44 (24.6%)		
Higher	18 (8.1%)	12 (6.7%)	7 (3.9%)	19 (10.6%)		
TSH-R Ab					0.67	0.411
Negative	34 (15.4%)	23 (12.8%)	11 (6.1%)	34 (19.0%)		
Positive	187 (84.6%)	106 (59.2%)	39 (21.8%)	145 (81.0%)		
Thyroglobulin antibody (TG Ab)					0.896	0.172
Negative	126 (57.0%)	82 (45.8%)	33 (18.4%)	115 (64.2%)		
Positive	95 (43.0%)	47 (26.3%)	17 (9.5%)	64 (35.8%)		
Thyroid peroxidase antibody (TPO Ab)					0.539	0.316
Negative	71 (32.1%)	46 (25.7%)	21 (11.7%)	67 (37.4%)		
Positive	150 (67.9%)	83 (46.4%)	29 (16.2%)	112 (62.6%)		
Thyroid stimulating immunoglobulin (TSI)					0.18	0.001
Negative	58 (26.2%)	19 (10.6%)	3 (1.7%)	22 (12.3%)		
Positive	163 (73.8%)	110 (61.5%)	47 (26.3%)	157 (87.7%)		
CAS	1.2 ± 1.2	2.4 ± 1.2	3.0 ± 1.4	2.5 ± 1.3	0.005	<0.001
NOSPECS	1.6 ± 1.3	3.0 ± 1.4	3.8 ± 1.5	3.2 ± 1.5	<0.001	<0.001
Proptosis					0.538	0.734
<15 mm	79 (35.7%)	42 (23.5%)	19 (10.6%)	61 (34.1%)		
15–18 mm	82 (37.1%)	45 (25.1%)	18 (10.1%)	63 (35.2%)		
>18 mm	60 (27.1%)	42 (23.5%)	13 (7.3%)	55 (30.7%)		
Type of eye deviation					<0.001	<0.001
Normal	175 (79.2%)	100 (55.9%)	11 (6.1%)	111 (62.0%)		
Horizontal	41 (18.6%)	19 (10.6%)	14 (7.8%)	33 (18.4%)		
Vertical	4 (1.8%)	9 (5.0%)	17 (9.5%)	26 (14.5%)		
Mixed	1 (0.5%)	1 (0.6%)	8 (4.5%)	9 (5.0%)		
Amount of eye deviation (°)	2.8 ± 6.7	3.3 ± 7.7	21.1 ± 19.2	8.3 ± 14.4	<0.001	<0.001
Central diplopia ^‡^					<0.001	<0.001
Absence	216 (97.7%)	110 (61.5%)	15 (8.4%)	125 (69.8%)		
Presence	5 (2.3%)	19 (10.6%)	35 (19.6%)	54 (30.2%)		
The Ratio of the Cross Sectional area to total Orbit (RCSO) ^†^						
Orbital fat	0.719 ± 0.085	0.712 ± 0.075	0.706 ± 0.093	0.710 ± 0.080	0.66	0.312
Superior rectus muscle	0.057 ± 0.020	0.059 ± 0.017	0.062 ± 0.023	0.060 ± 0.019	0.412	0.084
Medial rectus muscle	0.048 ± 0.015	0.049 ± 0.015	0.053 ± 0.019	0.050 ± 0.016	0.164	0.088
Inferior rectus muscle	0.057 ± 0.025	0.060 ± 0.020	0.070 ± 0.027	0.063 ± 0.022	0.025	0.03
Lateral rectus muscle	0.060 ± 0.024	0.062 ± 0.021	0.054 ± 0.026	0.060 ± 0.023	0.047	0.82
Total extraocular muscle ^††^	0.222 ± 0.084	0.230 ± 0.073	0.239 ± 0.095	0.233 ± 0.080	0.162	0.256

* RAI, radioactive iodine therapy. ^‡^ Diplopia within ≤30° of field of view measured by Binocular single vision test. ^†^ Ratio of the Cross-Sectional area to total Orbit (RCSO), cross sectional area measurement taken at the 4 mm behind the eyeball using computed tomography (CT). ^††^ Total extraocular muscle = superior rectus muscle + medial rectus muscle + inferior rectus muscle + lateral rectus muscle.

**Table 2:** The Score for Moderate to Severe type of Graves’ Orbitopathy Risk Prediction (SMSGOP) according to important variables.

Variables	Cut-Off	Score
Modified NOSPECS	0	0
	1–3	12
	4	20
	5≤	15
CAS	<3	0
	3	10
	4≤	15
Age (years)	<18	2
	18–28	0
	28–51	8
	51–64	12
	64≤	5
Central diplopia	Absence	0
	Presence	28
Thyroid stimulating immunoglobulin (TSI)	Negative	0
	Positive	8
RCSIRO *	I	0
	II	12
	III	15
	IV	18
Total		101

* RCSIRO, ratio of the cross sectional area of inferior rectus muscle to total orbit; I (<0.0296), II (0.0296–0.0395), III (0.0395–0.0764), IV (0.0764≤).

**Table 3:** The Score for Muscle predominant type of Graves’ orbitopathy Risk Prediction (SMGOP) according to important variables.

Variables	Cut-Off	Score
Amount of eye deviation (°)	<15	0
	15–45	3
	45≤	43
Age (years)	<32	0
	32–53	38
	53–64	41
	64≤	45
Central diplopia	Absence	0
	Presence	5
fT4	Lower	0
	Normal	5
	Higher	1
The interval between diagnosis of GD and GO (years)	<10	3
	10≤	0
Total		101

## Data Availability

The data presented in this study are available on request from the corresponding author. The data are not publicly available due to privacy.

## Supplementary Materials

The following supporting information can be downloaded at: https://www.mdpi.com/article/10.3390/jcm12072640/s1, Table S1: (A) Demographic for randomly split cohort for SMSGOP (training and validation cohort) (B) Demographic for randomly split cohort for SMGOP (training and validation cohort), Figure S1: (A) Number of Cases Versus Score Value on the Testing Cohort for SMSGOP (B) Observed Severity Rate Versus Score Value on the Testing Cohort for SMSGOP, Figure S2: (A) Number of Cases Versus Score Value on the Testing Cohort for SMGOP (B) Observed Severity Rate Versus Score Value on the Testing Cohort for SMGOP.
