# Supplementary material for: Application of an Interpretable Machine Learning for Estimating Severity of Graves’ Orbitopathy Based on Initial Finding
Source: J Clin Med. 2023 Apr 1;12(7):2640. doi: 10.3390/jcm12072640 (PMC10095042; doi:10.3390/jcm12072640)
Supplement: Supplementary file 1 [file jcm-12-02640-s001.zip › jcm-2242994-supplementary.pdf]

Supplemental Table S1. **(A)** Demographic for randomly split cohort for SMSGOP (training and validation cohort)

	**Training cohort (*n* = 320, 80%)**	**Validation cohort (*n* = 80, 20%)**	***p***
Age (years)	40.2 ± 13.8	37.7 ± 13.9	0.162
Gender			1
Female	241 (75.3%)	60 (75.0%)	
Male	79 (24.7%)	20 (25.0%)	
Duration of follow up for GD (months)	63.4 ± 47.7	56.5 ± 52.6	0.59
Duration of follow up for GO (months)	48.7 ± 42.1	43.7 ± 48.1	0.424
Interval between diagnosis of GD and GO (months)	18.3 ± 38.4	23.3 ± 52.1	0.425
Hypertension	34 (10.6%)	7 (8.8%)	0.773
Diabetes mellitus	11 (3.4%)	3 (3.8%)	1
Cerebrovascular disease	6 (1.9%)	1 (1.2%)	1
Autoimmune diseases	5 (1.6%)	3 (3.8%)	0.422
History of taking statin	36 (11.2%)	12 (15.0%)	0.465
Smoking behavior			0.444
Never smoker	285 (89.1%)	70 (87.5%)	
Ex-smoker	14 (4.4%)	2 (2.5%)	
Current smoker	21 (6.6%)	8 (10.0%)	
History of RAI *	9 (2.8%)	0 (0.0%)	0.273
History of thyroidectomy	10 (3.1%)	3 (3.8%)	1
fT3			0.823
Lower	5 (1.6%)	2 (2.5%)	
Normal	193 (60.3%)	49 (61.2%)	
Higher	122 (38.1%)	29 (36.2%)	
fT4			0.504
Lower	44 (13.8%)	9 (11.2%)	
Normal	141 (44.1%)	41 (51.2%)	
Higher	135 (42.2%)	30 (37.5%)	
Thyroid Stimulating Hormone (TSH)			0.76
Lower	226 (70.6%)	54 (67.5%)	
Normal	64 (20.0%)	19 (23.8%)	
Higher	30 (9.4%)	7 (8.8%)	
TSH-R Ab			0.172
Negative	59 (18.4%)	9 (11.2%)	
Positive	261 (81.6%)	71 (88.8%)	
Thyroglobulin antibody (TG Ab)			0.858
Negative	194 (60.6%)	47 (58.8%)	
Positive	126 (39.4%)	33 (41.2%)	
Thyroid peroxidase antibody (TPO Ab)			0.772
Negative	112 (35.0%)	26 (32.5%)	
Positive	208 (65.0%)	54 (67.5%)	
Thyroid stimulating immunoglobulin (TSI)			0.435
Negative	67 (20.9%)	13 (16.2%)	

Positive	253 (79.1%)	67 (83.8%)	
CAS	1.7 ± 1.4	2.0 ± 1.4	0.158
NOSPECS	2.3 ± 1.6	2.4 ± 1.5	0.673
Proptosis			0.612
<15 mm	111(34.7%)	27(33.8%)	
15–18 mm	122 (38.1%)	25 (31.2%)	
>18 mm	87 (27.2%)	28 (35.0%)	
Type of eye deviation			0.784
Normal	226 (70.6%)	60 (75.0%)	
Horizontal	61 (19.1%)	13 (16.2%)	
Vertical	24 (7.5%)	6 (7.5%)	
Mixed	9 (2.8%)	1 (1.2%)	
Amount of eye deviation (°)	5.5 ± 11.6	4.2 ± 9.4	0.306
Central diplopia ‡			0.549
Absence	275 (85.9%)	66 (82.5%)	
Presence	45 (14.1%)	14 (17.5%)	
The Ratio of the Cross-Sectional area to total Orbit (RCSO) †			
Orbital fat	0.717 ± 0.082	0.0708 ± 0.085	0.409
Superior rectus muscle	0.058 ± 0.020	0.060 ± 0.018	0.329
Medial rectus muscle	0.048 ± 0.015	0.051 ±0.017	0.222
Inferior rectus muscle	0.059 ± 0.024	0.062 ± 0.024	0.293
Lateral rectus muscle	0.060 ± 0.023	0.060 ± 0.024	0.852
Total extraocular muscle ††	0.225 ± 0.082	0.233 ± 0.083	0.256

* RAI, radioactive iodine therapy.

‡ Diplopia within ≤30° of field of view measured by Binocular single vision test.

† Ratio of the Cross-Sectional area to total Orbit (RCSO), cross sectional area measurement taken at the 4 mm behind the eyeball using computed tomography (CT).

†† Total extraocular muscle = superior rectus muscle + medial rectus muscle + inferior rectus muscle + lateral rectus muscle.

(B) Demographic for randomly split cohort for SMGOP (training and validation cohort)

	**Training cohort (*n* = 144)**	**Validation cohort (*n* = 35)**	***p***
Age (years)	42.6 ± 13.6	44.1 ± 15.2	0.584
Gender			0.706
Female	104 (72.2%)	27 (77.1%)	
Male	40 (27.8%)	8 (22.9%)	
Duration of follow up for GD (months)	61.4 ± 39.7	59.5 ± 43.2	0.47
Duration of follow up for GO (months)	49.2 ± 38.1	44.2 ± 50.1	0.398
Interval between diagnosis of GD and GO (months)	20.7 ± 45.3	30.6 ± 49.6	0.256
Hypertension	20 (13.9%)	5 (14.3%)	1
Diabetes mellitus	3 (2.1%)	1 (2.9%)	1
Cerebrovascular disease	2 (1.4%)	1 (2.9%)	1
Autoimmune diseases	3 (2.1%)	0 (0.0%)	0.899
History of taking statin	18 (12.5%)	9 (25.7%)	0.09
Smoking behavior			0.268
Never smoker	121 (84.0%)	30 (85.7%)	
Ex-smoker	9 (6.2%)	4 (11.4%)	

Current smoker	14 (9.7%)	1 (2.9%)	
History of RAI *	3 (2.1%)	2 (5.7%)	0.55
History of thyroidectomy	7 (4.9%)	2 (5.7%)	1
fT3			0.516
Lower	5 (3.5%)	0 (0.0%)	
Normal	92 (63.9%)	24 (68.6%)	
Higher	47 (32.6%)	11 (31.4%)	
fT4			0.605
Lower	22 (15.3%)	4 (11.4%)	
Normal	73 (50.7%)	21 (60.0%)	
Higher	49 (34.0%)	10 (28.6%)	
Thyroid Stimulating Hormone (TSH)			0.296
Lower	97 (67.4%)	19 (54.3%)	
Normal	32 (22.2%)	12 (34.3%)	
Higher	15 (10.4%)	4 (11.4%)	
TSH-R Ab			0.171
Negative	24 (16.7%)	10 (28.6%)	
Positive	120 (83.3%)	25 (71.4%)	
Thyroglobulin antibody (TG Ab)			0.049
Negative	87 (60.4%)	28 (80.0%)	
Positive	57 (39.6%)	7 (20.0%)	
Thyroid peroxidase antibody (TPO Ab)			0.186
Negative	50 (34.7%)	17 (48.6%)	
Positive	94 (65.3%)	18 (51.4%)	
Thyroid stimulating immunoglobulin (TSI)			0.645
Negative	19 (13.2%)	3 (8.6%)	
Positive	125 (86.8%)	32 (91.4%)	
CAS	2.7 ± 1.3	1.9 ± 1.3	0.003
NOSPECS	3.3 ± 1.5	2.7 ± 1.2	0.035
Proptosis			0.598
<15 mm	50 (34.7%)	12 (34.3%)	
15–18 mm	48 (33.3%)	12 (34.3%)	
>18 mm	46 (31.9%)	11 (32.4%)	
Type of eye deviation			0.347
Normal	90 (62.5%)	21 (60.0%)	
Horizontal	29 (20.1%)	4 (11.4%)	
Vertical	18 (12.5%)	8 (22.9%)	
Mixed	7 (4.9%)	2 (5.7%)	
Amount of eye deviation (°)	8.2 ± 14.9	8.4 ± 12.4	0.963
Central diplopia ‡			0.209
Absence	97 (67.4%)	28 (80.0%)	
Presence	47 (32.6%)	7 (20.0%)	
The Ratio of the Cross-Sectional area to total Orbit (RCSO) †			
Orbital fat	0.0710 ± 0.082	0.0714 ± 0.074	0.8
Superior rectus muscle	0.060 ± 0.019	0.059 ± 0.016	0.671
Medial rectus muscle	0.050 ± 0.016	0.050 ± 0.016	0.846
Inferior rectus muscle	0.063 ± 0.023	0.062 ± 0.020	0.82
Lateral rectus muscle	0.060 ± 0.023	0.059 ± 0.022	0.922
Total extraocular muscle ††	0.233 ± 0.081	0.230 ± 0.074	0.256

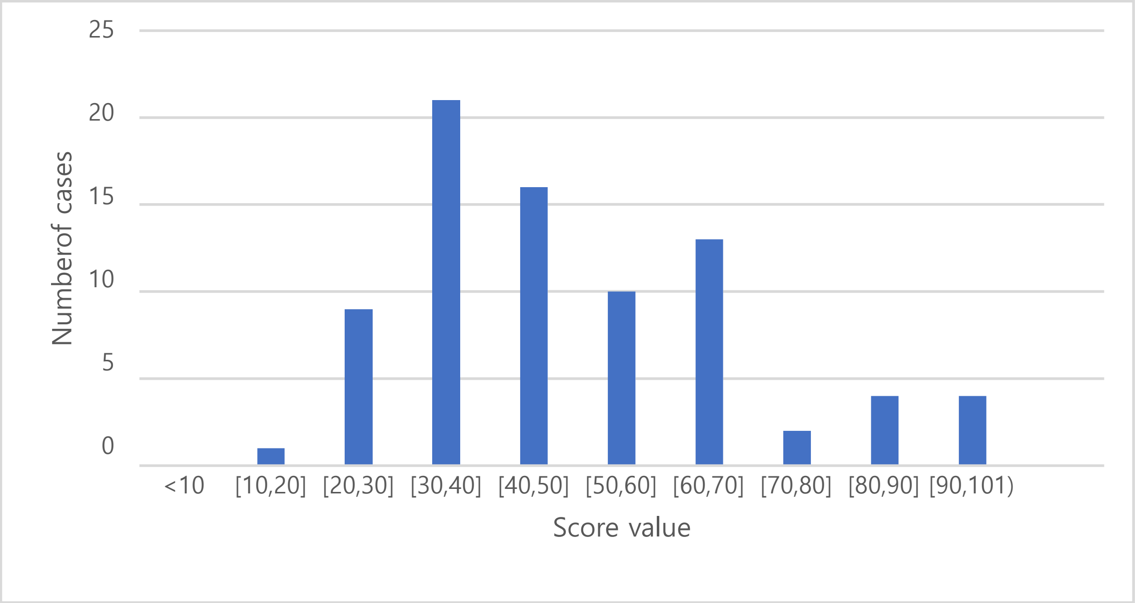

(**A**)

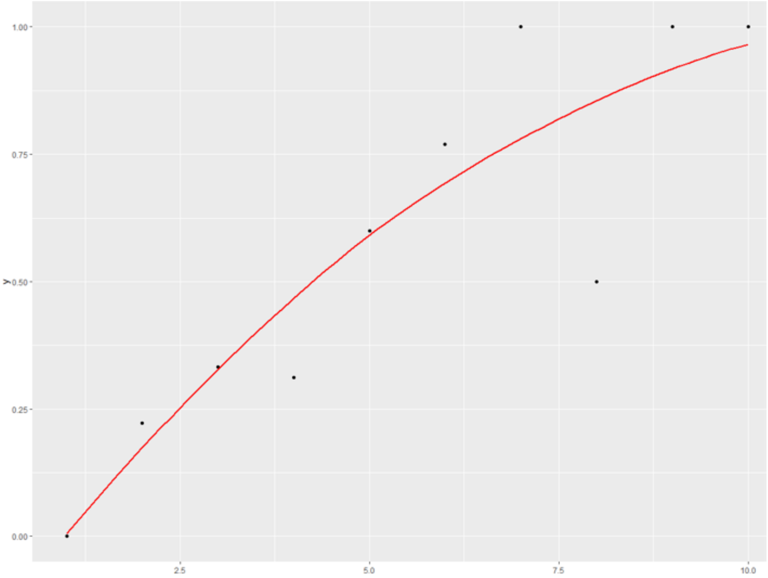

(**B**)

Supplemental Figure S1. (**A**) Number of Cases Versus Score Value on the Testing Cohort for SMSGOP (**B**) Observed Severity Rate Versus Score Value on the Testing Cohort for SMSGOP

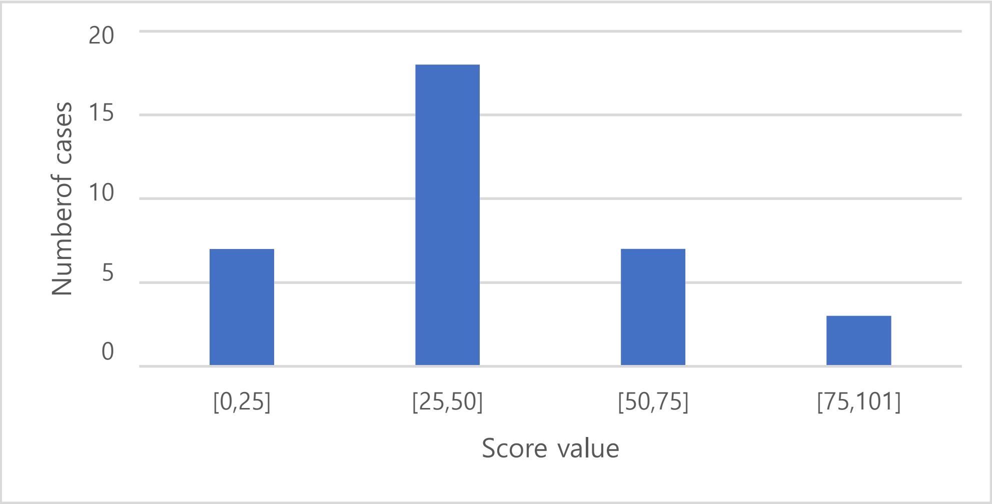

(**A**)

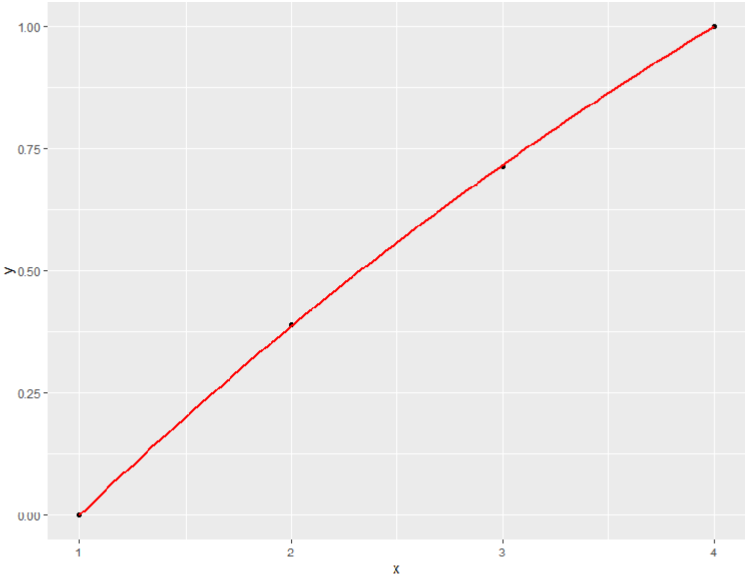

(**B**)

Supplemental Figure S2. (**A**) Number of Cases Versus Score Value on the Testing Cohort for SMGOP (**B**) Observed Severity Rate Versus Score Value on the Testing Cohort for SMGOP
